# Improved Success of Sparse Matrix Protein Crystallization Screening with Heterogeneous Nucleating Agents

**DOI:** 10.1371/journal.pone.0001091

**Published:** 2007-10-31

**Authors:** Anil S. Thakur, Gautier Robin, Gregor Guncar, Neil F. W. Saunders, Janet Newman, Jennifer L. Martin, Bostjan Kobe

**Affiliations:** 1 School of Molecular and Microbial Sciences, University of Queensland, St. Lucia, Brisbane, Queensland, Australia; 2 Institute for Molecular Bioscience and Australian Research Council (ARC) Special Research Centre for Functional and Applied Genomics, University of Queensland, St. Lucia, Brisbane, Queensland, Australia; 3 Molecular and Health Technologies, Commonwealth Scientific and Industrial Research Organization, Parkville, Victoria, Australia; Institute of Molecular and Cell Biology, Singapore

## Abstract

**Background:**

Crystallization is a major bottleneck in the process of macromolecular structure determination by X-ray crystallography. Successful crystallization requires the formation of nuclei and their subsequent growth to crystals of suitable size. Crystal growth generally occurs spontaneously in a supersaturated solution as a result of homogenous nucleation. However, in a typical sparse matrix screening experiment, precipitant and protein concentration are not sampled extensively, and supersaturation conditions suitable for nucleation are often missed.

**Methodology/Principal Findings:**

We tested the effect of nine potential heterogenous nucleating agents on crystallization of ten test proteins in a sparse matrix screen. Several nucleating agents induced crystal formation under conditions where no crystallization occurred in the absence of the nucleating agent. Four nucleating agents: dried seaweed; horse hair; cellulose and hydroxyapatite, had a considerable overall positive effect on crystallization success. This effect was further enhanced when these nucleating agents were used in combination with each other.

**Conclusions/Significance:**

Our results suggest that the addition of heterogeneous nucleating agents increases the chances of crystal formation when using sparse matrix screens.

## Introduction

Crystallization is considered to be a major bottleneck in the process of structure determination of macromolecules by X-ray crystallography. Crystallization is thought to occur in two steps: (i) nucleation and (ii) growth of nuclei to macroscopic crystals [1]. Fluctuations in protein concentration are assumed to be the driving force for protein crystallization. The term ‘nucleus’ refers to the smallest aggregate that is capable of spontaneous growth. In a typical protein crystallization experiment, crystals grow from a supersaturated aqueous solution by a process that involves homogenous nucleation. The nascent nuclei are in a state of equilibrium with the mother liquor, and when there is a sufficiently high supersaturation for nuclei to form, they can grow into crystals. Crystals can grow in the ‘metastable zone’ of the solubility diagram, but higher levels of saturation are needed for nucleation. The requirements for nucleation and growth of large, defect-free crystals are therefore disparate. If the protein concentration does not reach the required supersaturation level, the crystallization drop remains clear [2], [3].

In a popular approach to macromolecular crystallization, initial crystallization screening is carried out using sparse-matrix screens [4]. In such screens, a number of formulations suitable for crystallization are tested; however, protein and precipitant concentrations are not extensively sampled, therefore supersaturation conditions that support nucleation are often missed. Using small crystallization volumes (nano-crystallization), the likelihood of nucleation is further reduced [5], because the number of nuclei is proportional to the volume of crystallization solution [6].

An alternative mechanism to achieve nucleation is heterogeneous nucleation. Heterogeneous nucleation involves the introduction of a solid material termed the heterogeneous nucleating agent, nucleant or seed. Nucleation occurs on the surface of this material, which creates a higher local concentration of macromolecules, lowers the energy barrier for nucleation and bypasses the high kinetic barrier of spontaneous nucleation. A lower level of supersaturation is required under such circumstances for the nucleation step to occur, compared to homogenous nucleation.

Anecdotally, protein crystals have often been observed to grow on the surface of fortuitous impurities in the drop, such as dust particles and fibers. This has led to more systematic studies of the benefits of including heterogeneous nucleation agents in protein crystallization. The main lines of research have involved studies of epitaxic nucleants (epitaxial nucleation requires a correlation between the lattice of the heterogeneous nucleating agent and the nascent protein crystal; [7], [8]), lipid layers [9]–[11], natural surfaces such as whiskers, seeds and fibers [12], and fabricated substrates, made of silicon and other materials, displaying special surface properties such as terraces, steps and pores [13]–[19].

The mechanism of heterogeneous nucleation is complex, but the nucleation potential is mainly defined by the surface properties and chemical composition of the nucleating agent. For example, heterogeneous nucleation on fragments of human hair has been visualized by confocal fluorescent microscopy and atomic force microscopy, and the chemical and morphological properties of the nucleant surface have been investigated by treatment with chemicals [20]. The presence of keratin, but not lipids, was found to be essential for nucleation. Protein was observed to accumulate on sharp edges of the hair's cuticles, as previously observed for other surfaces [8], [12] and predicted by numerical simulations [21].

We reasoned that by including heterogeneous nucleating agents in a sparse matrix crystallization screen we could induce crystallization in a larger number of screen conditions. This idea is supported by recent studies using automated homogeneous microseeding to influence nucleation in crystallization screens [22]. We studied the effect of nine different potential nucleating agents and their combinations on the crystallization of ten different proteins in a sparse matrix screen. We observed that several nucleating agents induced crystallization in conditions that did not yield crystals in the absence of the nucleating agent. Four nucleating agents (dried seaweed, horse hair, cellulose and hydroxyapatite) showed considerable positive effects on crystallization success. The most efficient single nucleating agent was found to be dried seaweed, but the best results were obtained by using multiple nucleants simultaneously in the drop. Our results suggest that the addition of heterogeneous nucleating agents is a simple method for increasing the chances of crystal formation when using sparse matrix crystallization screens.

## Results and Discussion

### Experimental design

The potential heterogeneous nucleating agents were chosen to cover a diverse range of easily available materials and surface types with properties that might be favorable for nucleation, based on past literature reports [12], [18]. Proteins adsorb onto surfaces by a combination of hydrophobic and electrostatic interactions, and the precise nature of the interaction will depend on both the protein and the surface. Often, adsorption induces partial denaturation of the protein, although this effect is less pronounced on neutral hydrophilic surfaces [23]. Ten commercially available proteins, which are known to crystallize, were chosen to cover a wide range of pIs and molecular masses. Crystal Screen HT [4] was chosen as the sparse matrix crystallization screen, because it is one of the first and most widely used commercially available crystallization screens (for the list of formulations, see Supplementary Table S1). The hanging drop vapor diffusion technique was used in these experiments, and the crystallizations were set up and monitored in 96-well plates using robotic equipment, following a standard high-throughput crystallization protocol. In the absence of nucleating agents, crystals were observed in three to six out of the 96 sparse matrix formulations for each of the proteins studied (Table 1, Supplementary Table S2); the experiment in the absence of nucleating agents represents the control experiment.

**Table 1:** Summary of the effects of heterogenous nucleating agents.

Protein	Control (no nucleant)	Fumed silica		CM Sephadex		Sand		Titanium(IV) oxide		Glass wool		Hydroxy-apatite		Cellulose		Horse hair		Dried seaweed		Combination of 9 nucleants		Combination of 4 nucleants	
	Total	+	−	+	−	+	−	+	−	+	−	+	−	+	−	+	−	+	−	+	−	+	−
Lysozyme	5	1	3	0	1	0	0	1	1	0	0	1	0	2	0	1	0	4	0	7	3	7	0
Pepsin	3	0	1	1	2	0	0	1	0	0	0	2	1	0	0	2	0	2	0	4	1	ND	
Trypsin	5	1	2	0	2	0	0	0	1	1	0	2	1	3	0	2	0	3	0	5	1	ND	
Glucose Isomerase	4	0	3	0	1	0	0	0	0	0	0	1	2	2	1	1	0	2	0	3	1	ND	
Ribonuclease A	6	0	4	1	2	0	0	1	1	0	0	2	2	2	0	3	1	2	1	3	2	ND	
Myoglobin	4	0	3	0	1	0	1	0	1	0	0	1	1	2	0	1	0	2	0	4	0	ND	
α-lactalbumin	5	1	1	1	0	0	0	0	1	0	0	1	1	1	0	2	1	3	0	0	0	ND	
Catalase	5	2	2	0	1	0	0	0	0	0	0	2	1	1	1	3	1	1	0	6	2	ND	
Xylanase	5	1	1	0	1	0	0	1	1	0	0	3	1	0	0	3	1	2	1	6	1	ND	
Thaumatin	4	2	2	0	1	0	0	1	0	0	0	2	0	3	1	2	0	2	1	5	1	ND	
Sum over all proteins	46	8	22	3	12	0	1	5	6	1	0	17	10	16	3	20	4	23	3	43	12	ND	
Total effect (crystallization hits)		−14		−9		−1		−1		1		7		13		16		20		31		ND	
Total effect (%)		−30		−20		−2		−2		2		15		28		35		43		67		ND	

For each protein/heterogeneous nucleating agent pair and the sum over all proteins, the number of new and missing crystallization conditions (“+” and “−”, respectively) is shown, relative to the no-nucleant control. For the no-nucleant control, the total number of crystallization hits is shown. The total effect of the nucleating agent, relative to the no-nucleant control, is shown in the last two rows. “ND”, not determined.

### Effect of nucleating agents

**Figure 1:**
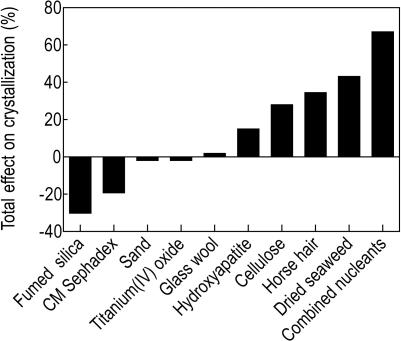
Effect of heterogenous nucleating agents. The height of the bar represents the relative difference between the number of crystals observed in the presence and the absence of a heterogeneous nucleating agent, summed over all the proteins tested. Details of the data are presented in Table 1 and Supplementary Table S2. “Combined nucleants” refers to a mixture of all 9 nucleants.

**Figure 2:**
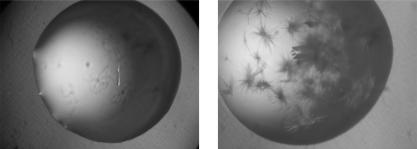
Example of a positive effect of a heterologous nucleating agent on crystallization. (A) Control crystallization drop with no heterogeneous nucleating agent added (glucose isomerase, condition F4: 2% w/v polyethyleneimine, 0.1 M sodium citrate pH 5.6, 0.5 M sodium chloride). (B) Crystallization drop with identical crystallization conditions as in A, but with horse hair added as a heterogeneous nucleating agent.

To assess the effect of heterogeneous nucleating agents, these were added to the protein solution and crystallization plates set up in an identical fashion to the control experiment. Out of the nine individual nucleants studied, four had a considerable positive effect on the crystal nucleation, compared to the control (Figure 1, Table 1, Supplementary Table S2). An example of a crystallization experiment where the nucleant had a positive effect is shown in Figure 2. For the proteins tested, dried seaweed, horse hair, hydroxyapatite and cellulose produced crystals in 23, 20, 17 and 16 novel conditions respectively, compared to the no-nucleant control. Although these nucleants also inhibited the growth of crystals in some conditions, the overall effects were positive: 43%, 35%, 28% and 15% more crystallization hits compared to the control for dried seaweed, horse hair, cellulose and hydroxyapatite, respectively. By contrast, fumed silica produced crystals in 8 novel conditions, but inhibited crystal growth in 22, resulting in an overall negative effect. Carboxymethyl (CM) Sephadex also had an overall negative effect. Sand, glass wool and titanium(IV) oxide had no major effect on crystallization.

Combining all nine nucleants with each other had a higher overall positive effect than for any individual nucleant; the combined nucleants induced crystal growth in 43 new conditions and produced 67% more crystallization hits than the experiment in the absence of any nucleant. After identifying the four best nucleants from the individual nucleant experiments, we combined these four (dried seaweed, horse hair, cellulose and hydroxyapatite) and repeated the experiment on lysozyme (Table 1, Supplementary Table S2). The results of this experiment showed a further improvement over the combination of the nine nucleants.

### Effect of the amount of nucleating agent

All the initial experiments were performed using a set amount of nucleating agent, chosen somewhat arbitrarily as the largest amount of nucleant that allowed unobstructed visualization of crystals under the microscope (0.5 µg finely ground nucleant per µl of protein, equivalent to 0.25 µg nucleant per µl of the crystallization droplet). To examine the effect of the quantity of nucleating agent, we chose one crystallization condition (lysozyme/dried seaweed, condition D3: 0.1 M Hepes (pH 7.5), 2% PEG 400, 2 M ammonium sulfate) and set up drops with diminishing amounts of nucleant (6 replicate drops for each amount of nucleant). Crystals appeared in 92%, 80% and 33% of the drops containing 1, 1/25 and 1/50 of the initial amount of the nucleant, respectively. The results indicate that reducing the amount of nucleating agent from the original value we used reduces the chances of successful nucleation.

### Assessment of reproducibility

Nucleation and crystallization occur as a result of random association events and are influenced by a number of different factors. It is therefore important to establish the reproducibility of the effect of heterogeneous nucleating agents. However, due to the scope of the initial experiment to establish the effect of different nucleants (10 protein variables and 11 nucleating agent variables), resulting in over one hundred 96-well crystallization plates and over 10,000 crystallization drops, it was not feasible to set up replicates of the whole experiment. Instead, we performed duplicate experiments for selected conditions. Based on the results of the original crystallization screen experiments, eight conditions that showed a positive nucleation effect (i.e. that led to crystal formation in conditions that did not produce crystals in the absence of nucleants) were chosen for each protein (see also Materials and Methods), and twelve replicates for each condition were set up. A similar control plate was set up without the nucleant. The results are summarized in Table 2. If there were 100% reproducibility, 12 out of the 12 drops should produce crystals in the presence of the nucleating agent, and none of the 12 control drops should produce crystals. The results show no crystals in any of the drops without the nucleant, and a mean of 9.1±1.4 drops with crystals in the presence of nucleants (Table 2). The analysis of the reproducibility for individual proteins revealed no substantial variations; the analysis for individual nucleating agents, however, revealed a lower reproducibility for titanium(IV) oxide and CM Sephadex (Table 2).

**Table 2:** Reproducibility of crystal formation.

*Protein*	*Number of replicates*	*Mean±standard deviation per protein/nucleant combination*
Lysozyme	96	9.4±1.4
Pepsin	96	9.2±1.3
Trypsin	96	9.2±1.9
Glucose isomerase	96	8.6±1.2
RNase A	96	8.6±1.6
Myoglobin	96	8.9±1.4
α-Lactalbumin	96	9.4±1.4
Catalase	96	9.2±1.3
Xylanase	96	9.2±1.3
Thaumatin	96	9.2±1.3
*Nucleant*		
Fumed silica	96	8.1±1.1
CM Sephadex	36	7.7±0.6
Sand	-	-
Titanium(IV) oxide	48	6.7±0.5
Glass wool	-	-
Hydroxyapatite	168	9.2±1.1
Cellulose	168	9.3±1.0
Horse hair	204	9.9±1.6
Dried seaweed	228	9.6±1.2
Overall	864	9.1±1.4

“-”, not applicable. Mean and standard deviation were calculated using Microsoft Excel. Twelve replicates were set up for each protein/nucleant combination tested, therefore the maximum possible number for the mean per protein/nucleant combination is 12. The no-nucleant control experiment to test reproducibility produced no crystals in any of the drops (therefore the mean±standard deviation per protein/nucleant combination for the no-nucleant control is 0. 0±0.0 in all cases).

### Comparison of nucleating agents

Among the heterogeneous nucleating agents we studied, dried seaweed emerged as the most successful nucleant, triggering crystallization in new conditions for every one of the proteins tested, and inhibiting crystallization in only very few cases, as compared with the control. Notably positive results were also obtained with horse hair and cellulose. Hydroxyapatite led to new crystallization conditions for all the proteins tested, but also inhibited crystallization in a larger number of cases, although the overall effect was positive. By contrast, while fumed silica and CM Sephadex identified some new crystallization conditions, they inhibited many more, resulting overall in a negative effect. Other nucleants tested had a smaller overall effect, with glass wool and sand displaying no major effects on crystallization.

Interestingly, the conditions identified as supporting crystallization only with the addition of nucleant tended to be unique to a given nucleant. The less successful nucleants identified few if any unique crystallization conditions. Consistent with these observations, combining the different nucleating agents with each other had a stronger positive effect than any individual nucleant. Our data do not show clear evidence of a synergistic effect; the effect of combining the nucleants with each other is most likely cumulative.

An analysis of the conditions in which the nucleants induced new crystallizations reveals some interesting trends. For example, dried seaweed and hydroxyapatite seem to preferentially cause nucleation in formulations containing PEG as precipitant, whereas horse hair was more successful in conditions that did not contain PEG. Interestingly, most of the inhibition caused by dried seaweed also occurred in conditions containing PEG as precipitant. Validation of the observed trends will require further testing.

Our results are not surprising, as several heterogeneous nucleating agents used here have been found previously by others to have positive effects. For example, horse hair and dried seaweed have been reported previously to promote nucleation of lysozyme, glucose isomerase, trypsin and malonyl coenzyme A-acyl carrier protein transacylase [12]. Similarly, porous glass substrate facilitated the nucleation of hen egg-white lysozyme, thaumatin and apoferritin at low supersaturation [18], and human hair was found to induce crystallization under conditions where spontaneous nucleation did not occur, and at lower protein concentrations than required for homogeneous crystallization [20]. Our results support previous conclusions that the nucleation potential depends on the surface properties and chemical composition of the nucleating agent. Sand and glass wool may not have an irregular enough surface or the appropriate chemical composition to display any major effects on nucleation. However, it may also be that for these nucleants we simply did not effectively transfer the nucleants to the protein samples.

### Addition of nucleants to the crystallization experiment

We chose to add the nucleating agent to the protein solution before combining protein and reservoir solutions in the drop, as this appeared to be the simplest way to add the nucleating agent when setting up sparse matrix screens. The results show that this approach achieves reasonable reproducibility. With different automation, different techniques might be more appropriate [22]. If larger drops are used, then it might become possible to add the nucleant as a separate component to the crystallization droplet.

### Effect of vapor diffusion technique

We also tested if our approach can be used with the sitting drop vapor diffusion technique, as sitting drop experiments are becoming the standard in many medium- and high-throughput crystallization laboratories. We saw a similar positive effect with both hanging drop and sitting drop approaches for a lysozyme/dried seaweed experiment (Supplementary Table S2). Thorough comparison of the differential effects of heterogeneous nucleating agents in hanging drop and sitting drop experiments will require further studies.

### Crystal optimization

One may choose to optimize any crystallization hit (obtained by screening in the presence of heterogeneous nucleating agents) either with or without the nucleant. We set up a crystallization experiment for lysozyme (in the absence of the nucleating agent) under a condition that only yielded crystals in the presence of dried seaweed as the nucleating agent (condition C6: 0.2 M ammonium sulfate, 30% PEG 8000). We then varied the precipitant concentration (in the absence of the nucleating agent). At a higher precipitant concentration (PEG concentration of 32–34%), crystals appeared in the absence of the nucleating agent. This result is consistent with a model suggesting that when crystallization only occurs in the presence of the heterogeneous nucleating agent, it likely occurs in the metastable zone of the phase diagram. In such cases, crystallization is likely achievable in the absence of nucleating agent if protein or precipitant concentrations were increased.

Alternatively, a crystallization hit obtained by screening in the presence of heterogeneous nucleating agents provides material for traditional seeding approaches using homo-nucleation [24]. As a further alternative, crystal optimization could be pursued in the presence of nucleating agent, with the amount of nucleating agent used as a variable. We are not aware of general adverse effects of the nucleating agent on diffraction quality; indeed, crystals grown at a lower level of supersaturation may have improved diffraction quality [25]. In the future, nano-sized crystals forming on the surface of heterogeneous nucleating agents could also be used for structure determination using electron crystallography, removing the need for optimization [20].

### Conclusions

The use of heterogeneous nucleating agents has much broader utility than traditional seeding techniques used in protein crystallization, such as macroseeding, microseeding and streak seeding [24]. With these seeding techniques, protein crystals or crystal seeds have to be produced first, and these dissolve if introduced in the undersaturated phase. By contrast, insoluble heterogeneous nucleating agents do not dissolve if placed in the undersaturated phase.

We compared several heterogeneous nucleating agents and showed that the addition of some of these provides a simple method to increase the chances of crystal formation when using sparse matrix crystallization screens. The most successful nucleating agents consisted of pulverized dried seaweed, horse hair, cellulose and hydroxyapatite, all widely available materials. Although mixtures of nucleants perform better than individual nucleants, it may be advantageous to set up crystallization screens in the presence of individual nucleants as well; individual nucleants often yielded crystals under unique crystallization conditions. We recognize that we have screened only a limited range of materials, and further studies may uncover heterogeneous nucleating agents with even better properties when used to increase the success of crystallization screening.

## Materials and Methods

### Materials

The proteins catalase (Cat. No. C-3155), myoglobin (Cat. No. M0630), ribonuclease A (Cat. No. R4875), pepsin (Cat. No. P7000), thaumatin (Cat. No. T7638), trypsin (Cat. No. T8003), xylanase (Cat. No. X2753) and α-lactalbumin (Cat. No. L5385) were purchased from Sigma-Aldrich, Missouri, U.S.A. Lysozyme (Cat. No. 837059) was obtained from Roche Applied Sciences, Indianapolis, U.S.A, and glucose isomerase (Cat. No. HR7-100) from Hampton Research, California, U.S.A. The proteins were dissolved in 25 mM Tris-HCl (pH 7.0) except for glucose isomerase, which was dialyzed into 10 mM Tris (pH 7.0) and 1 mM magnesium chloride. All proteins were used at a concentration of 10 mg/ml. The proteins were filtered through a 0.22 µm filter (Millipore, Carringtwohill Co. Cork, Ireland).

The heterogeneous nucleants titanium(IV) oxide (Cat. No. 634662), carboxymethyl (CM) Sephadex (Cat. No. C50120), cellulose (Cat. No. 310697), hydroxyapatite (Cat. No. 289396), fumed silica (Cat. No. S5130) and Pyrex fiber glass wool (Cat. No. CLS3950) were obtained from Sigma-Aldrich, Missouri, U.S.A. Horse hair was obtained from a local violin shop, sand was obtained from a local beach, and green seaweed (genus name *Ulva*, local name hai-tsai) was purchased from a local Asian grocery store in the form of fresh seaweed (imported by Jyie Nung Holdings, Brisbane, Australia).

### Preparation of heterogeneous nucleating agents

Seaweed was washed with milliQ water to remove any surface contaminants and dried in a drying oven at 60°C for 36 h. For each of the heterogeneous nucleating agents, 1 g was placed into a mortar, the mortar filled to one-quarter level with liquid nitrogen, and the material pulverized using a pestle, until a fine powder was obtained. The combination of heterogenous nucleants with each other was prepared by grinding the nucleants individually to a fine powder and then mixing an equal amount (0.5 mg) of each nucleant in an Eppendorf tube.

### Addition of heterogeneous nucleating agent to the protein solution

The heterogeneous nucleating agent was added in the ratio of 0.5 µg to 1 µl of the protein solution and was mixed gently by tapping the tube. The solution was stored on ice and used within 30 min.

### Crystallization

Crystal Screen HT sparse matrix crystallization screen (Hampton Research, California, U.S.A) was used in this study. In order to minimize the contamination from any fortuitous substances such as dust particles and fibers, crystallization plates were removed from their plastic covers just prior to setting up the experiments and sealed with sealing tape (Qiagen Inc, California, U.S.A.) immediately after 96 sparse matrix crystallization conditions were dispensed into the plate, and stored at 4°C until further use. One hundred µl (50 µl in the case of sitting drops) of crystallization condition was dispensed in each well of the 96-well plate. For hanging drop experiments, we used 96-well plates from TPP (MIDSCI, Missouri, U.S.A) and Viewdrop 96-well plate seals from Millennium Science (Victoria, Australia). For sitting drop experiments, Greiner low profile 96-well plates were used. In both cases, crystallization drops containing 100 nl protein solution were combined with 100 nl of reservoir solution using a Mosquito robot (TTP LabTech, Melbourn, UK) at room temperature. All plates were incubated at 20°C.

To assess the reproducibility of crystallization experiments, eight conditions that showed a positive nucleation effect (i.e. that led to crystal formation in conditions that did not produce crystals in the absence of nucleants) were chosen for each protein based on the results of the original crystallization screen experiments. Twelve replicates for each condition were set up across a row of a 96-well plate. A similar control plate was set up without the nucleant. In the case of glucose isomerase and myoglobin, only six conditions with a positive nucleation effect were available, therefore two of the conditions were repeated to fill the 96-well plate.

### Imaging

Crystallisation experiments were imaged using a Crystal Monitor workstation (Emerald Biosystems, Washington, USA) on days 1, 3, 7 and 14. The data from day 7 were used in the analysis of results. All crystalline objects were counted as crystallization hits, as assessed by straight edges and using polarized light.

## Supporting Information

Table S1 Formulations in Crystal Screen HT
(0.08 MB PDF)

Table S2 All successful crystallizations
(0.08 MB PDF)
