# Supplementary material for: Improved Success of Sparse Matrix Protein Crystallization Screening with Heterogeneous Nucleating Agents
Source: PLoS One. 2007 Oct 31;2(10):e1091. doi: 10.1371/journal.pone.0001091 (PMC2034409; doi:10.1371/journal.pone.0001091)
Supplement: Table S1 — Formulations in Crystal Screen HT (0.08 MB PDF) [file pone.0001091.s001.pdf]

Table S1
Formulations in Crystal Screen HT.

Number	Precipitant	Buffer (100 mM)	Additive
A1	30% v/v MPD	0.1 M Na Acetate pH 4.6	0.02 M calcium chloride
A2	0.4 M Na/K tartrate		
A3	0.4 M ammonium phosphate		
A4	2.0 M ammonium sulphate	0.1 M Tris HCl pH 8.5	
A5	30% v/v MPD	0.1 M Na Hepes pH 7.5	0.2 M sodium citrate
A6	30% w/v PEG 4000	0.1 M Tris HCl pH 8.5	0.2 M magnesium chloride
A7	1.4 M sodium acetate	0.1 M Na cacodylate pH 6.5	
A8	30% v/v isopropanol	0.1 M Na cacodylate pH 6.5	0.2 M sodium citrate
A9	30% w/v PEG 4000	0.1 M Na citrate pH 5.6	0.2 M ammonium acetate
A10	30% w/v PEG 4000	0.1 M Na acetate pH 4.6	0.2 M ammonium acetate
A11	1.0 M ammonium phosphate	0.1 M Na citrate pH 5.6	
A12	30% v/v isopropanol	0.1 M Na Hepes pH 7.5	0.2 M magnesium chloride
B1	30% v/v PEG 400	0.1 M Tris HCl pH 8.5	0.2 M sodium citrate
B2	28% v/v PEG 400	0.1 M Na Hepes pH 7.5	0.2 M calcium chloride
B3	30% w/v PEG 8000	0.1 M Na cacodylate pH 6.5	0.2 M ammonium sulphate
B4	1.5 M lithium sulphate	0.1 M Na Hepes pH 7.5	
B5	30% w/v PEG 4000	0.1 M Tris HCl pH 8.5	0.2 M lithium sulphate
B6	20% w/v PEG 8000	0.1 M Na cacodylate pH 6.5	0.2 M magnesium acetate
B7	30% v/v isopropanol	0.1 M Tris HCl pH 8.5	0.2 M ammonium acetate
B8	25% w/v PEG 4000	0.1 M Na acetate pH 4.6	0.2 M ammonium sulphate
B9	30% v/v MPD	0.1 M Na cacodylate pH 6.5	0.2 M magnesium acetate
B10	30% w/v PEG 4000	0.1 M Tris HCl pH 8.5	0.2 M sodium acetate
B11	30% v/v PEG 400	0.1 M Na Hepes pH 7.5	0.2 M magnesium chloride
B12	20% v/v isopropanol	0.1 M Na acetate pH 4.6	0.2 M calcium chloride
C1	1.0 M sodium acetate	0.1 M imidazole pH 6.5	
C2	30% v/v MPD	0.1 M Na citrate pH 5.6	0.2 M ammonium acetate
C3	20% v/v isopropanol	0.1 M Na Hepes pH 7.5	0.2 M sodium citrate
C4	30% w/v PEG 8000	0.1 M Na cacodylate pH 6.5	0.2 M sodium acetate
C5	0.8 M Na/K tartrate	0.1M Na Hepes pH 7.5	
C6	30% w/v PEG 8000		0.2 M ammonium sulphate
C7	30% w/v PEG 4000		0.2 M ammonium sulphate
C8	2.0 M ammonium sulphate		
C9	4.0 M sodium formate		
C10	2.0 M sodium formate	0.1 M Na acetate pH 4.6	
C11	0.8 M Na/K phosphate	0.1 M Na Hepes pH7.5	
C12	8% w/v PEG 8000	0.1 M Tris HCl pH 8.5	
D1	8% w/v PEG 4000	0.1 M Na acetate pH 4.6	
D2	1.4 M sodium citrate	0.1 M Na Hepes pH 7.5	
D3	2% v/v PEG 400, 2.0 M ammonium sulphate	0.1 M Na Hepes pH 7.5	
D4	20% v/v isopropanol, 20% w/v PEG 4000	0.1 M Na citrate pH 5.6	
D5	10% v/v isopropanol, 20% w/v PEG 4000	0.1 M Na Hepes pH 7.5	
D6	20% w/v PEG 8000		0.05 M potassium phosphate
D7	30% w/v PEG 1500		
D8	0.2 M magnesium formate		
D9	18% w/v PEG 8000	0.1 M Na cacodylate pH 6.5	0.2 M zinc acetate
D10	18% w/v PEG 8000	0.1 M Na cacodylate pH 6.5	0.2 M calcium acetate
D11	2.0 M ammonium sulphate	0.1 M Na acetate pH 4.6	
D12	2.0 M Ammonium phosphate	0.1 M Tris HCl pH 8.5	
E1	10% w/v PEG 6000		2.0 M sodium chloride
E2	0.01 M Hexacethyltrimethylammonium bromide (CTAB)		0.5 M sodium chloride, 0.01 M magnesium chloride
E3	25% v/v ethylene glycol		
E4	35% v/v dioxane		
E5	5% v/v isopropanol		2.0 M ammonium sulphate
E6	1.0M imidazole pH 7.0		
E7	10% w/v PEG 1000, 10% w/v PEG 8000		
E8	10% v/v ethanol		1.5 M sodium chloride
E9	2.0 M sodium chloride	0.1 M Na acetate pH 4.6	
E10	30% v/v MPD	0.1 M Na acetate pH 4.6	0.2 M sodium chloride
E11	1.0 M 1,6 hexanediol	0.1 M Na acetate pH 4.6	0.01 M cobalt chloride
E12	30% v/v PEG 400	0.1 M Na acetate pH 4.6	0.1M cadmium chloride
F1	30% w/v PEG MME 2000	0.1 M Na acetate pH 4.6	0.2 M ammonium sulphate
F2	2.0 M ammonium sulphate	0.1 M Na citrate pH 5.6	0.2M K/Na tartrate
F3	1.0 M lithium sulphate	0.1 M Na citrate pH 5.6	0.5M ammonium sulphate
F4	2% w/v polyethyleneimine	0.1 M Na citrate pH 5.6	0.5 M sodium chloride
F5	35% v/v tert-butanol	0.1 M Na citrate pH 5.6	
F6	10% v/v Jeffamine M-600	0.1 M Na citrate pH 5.6	0.01 M ferric chloride
F7	2.5 M 1,6 hexanediol	0.1 M Na citrate pH 5.6	
F8	1.6 M magnesium sulphate	0.1 M MES pH 6.5	
F9	2.0 M sodium chloride	0.1 M MES pH 6.5	0.2 M Na/K phosphate
F10	12% w/v PEG 20,000	0.1 M MES pH 6.5	
F11	10% v/v dioxane	0.1 M MES pH 6.5	1.6 M ammonium sulphate

F12	30% v/v Jeffamine M-600	0.1 M MES pH 6.5	0.05 M cesium chloride
G1	1.8 M ammonium sulphate	0.1 M MES pH 6.5	0.01 M cobalt chloride
G2	30% w/v PEG MME 5000	0.1 M MES pH 6.5	0.2 M ammonium sulphate
G3	25% v/v PEG MME 550	0.1 M MES pH 6.5	0.01 M zinc sulphate
G4	1.6 M sodium citrate pH 6.5		
G5	30% v/v MPD	0.1 M Hepes pH 7.5	0.5 M ammonium sulphate
G6	10% w/v PEG 6000, 5% v/v MPD	0.1 M Hepes pH 7.5	
G7	20% v/v Jeffamine M-600	0.1 M Hepes pH 7.5	
G8	1.6 M ammonium sulphate	0.1 M Hepes pH 7.5	0.1 M sodium chloride
G9	2.0 M ammonium formate	0.1 M Hepes pH 7.5	
G10	1.0 M sodium acetate	0.1 M Hepes pH 7.5	0.05 M cadmium sulphate
G11	70% v/v MPD	0.1 M Hepes pH 7.5	
G12	4.3 M sodium chloride	0.1 M Hepes pH 7.5	
H1	10% w/v PEG 8000, 8% v/v ethylene glycol	0.1 M Hepes pH 7.5	
H2	20% w/v PEG 10,000	0.1 M Hepes pH 7.5	
H3	3.4 M 1,6 hexanediol	0.1 M Tris pH 8.5	0.2 M magnesium chloride
H4	25% v/v tert-butanol	0.1 M Tris pH 8.5	
H5	1.0 M lithium sulphate	0.1 M Tris pH 8.5	0.01 M nickel (II) chloride
H6	12% v/v glycerol	0.1 M Tris pH 8.5	1.5 M ammonium sulphate
H7	50% v/v MPD	0.1 M Tris pH 8.5	0.2 M ammonium phosphate
H8	20% v/v ethanol	0.1 M Tris pH 8.5	
H9	20% w/v PEG MME 2000	0.1 M Tris pH 8.5	0.01 M nickel (II) chloride
H10	20% w/v PEG MME 550	0.1 M Bicine pH 9.0	0.1 M sodium chloride
H11	2.0 M magnesium chloride	0.1 M Bicine pH 9.0	
H12	10% w/v PEG 20,000	0.1 M Bicine pH 9.0	2% v/v dioxane
