# Supplementary material for: Improved Success of Sparse Matrix Protein Crystallization Screening with Heterogeneous Nucleating Agents
Source: PLoS One. 2007 Oct 31;2(10):e1091. doi: 10.1371/journal.pone.0001091 (PMC2034409; doi:10.1371/journal.pone.0001091)
Supplement: Table S2 — All successful crystallizations (0.08 MB PDF) [file pone.0001091.s002.pdf]

Table S2

All successful crystallizations. The formulations in Crystal Screen HT leading to crystal formation are indicated as labeled in Supplementary Table S2, (New crystallization hits in the presence of nucleant are shown in bold.

Protein	No nucleant	Fumed silica	CM Sephadex	Sand	Titanium (IV) oxide	Glass wool	Hydroxy-apatite	Cellulose	Horse hair	Dried seaweed	Combination of 9 nucleants	Combination of 4 nucleants	No nucleant (sitting drop)	Dried seaweed (sitting drop)
**Lysozyme**	A9, C10, D9, E9, H3	**D12**, C10, H3	C10, D9, E9, H3	A9, C10, D9, E9, H3	**F1**, A9, D9, E9, H3	A9, C10, D9, E9, H3	**B5**, A9, C10, D9, E9, H3	**F1, E3**, A9, C10, D9, E9, H3	**E8**, A9, C10, D9, E9, H3	**C6, C7, D3, D12,** A9, C10, D9, E9, H3	**C6, C7, D3, D12**, **E3, E8, F1,** C10, H3	**B5, C6, C7, D3, D12**, **E8, F1,** A9, C10, D9, E9, H3	A9, C10, D9, E1, E9, F9	**B1, C11, D3**, A9, C10, D9, E1, E9, F9
**Pepsin**	B5, C3, H2	B5, *C3*	**D4**, H2	B5, C3, H2	**D2**, B5, C3, H2	B5, C3, H2	**D1, D5**, B5, H2	B5, C3, H2	**A3, A5**, B5, C3, H2	**G4, G7**, B5, C3, H2	**D2, D5**, **G4, G7,** B5, H2			
**Trypsin**	A5, C3, D2, F2, F5	**D5**, A5, C3, F5	A5, C3, F5	A5, C3, D2, F2, F5	A5, C3, D2, F2	**D5**, A5, C3, D2, F2, F5	**A6, A10**, A5, C3, D2, F2	**G3, G7, H2**, A5, C3, D2, F2, F5	**H5, H7**, A5, C3, D2, F2, F5	**D5, F3, F6**, A5, C3, D2, F2, F5	**D5, G3, H5, H7, F3**, A5, C3, D2, F5			
**Glucose Isomerase**	B9, C11, D5, E5	B9	B9, D5, E5	B9, C11, D5, E5	B9, C11, D5, E5	B9, C11, D5, E5	**F3**, B9, C11	**C3, C7**, B9, C11, E5	**F4**, B9, C11, D5, E5	**H9, H12**, B9, C11, D5, E5	**F4, H9, H12**, B9, D5, E5			
**RNase A**	A4, A7, B5, B6, F2, G2	F2, G2	**A5**, A4, A7, B5, B6	A4, A7, B5, B6, F2, G2	**A5**, A4, A7, B5, F2, G2	A4, A7, B5, B6, F2, G2	**B2, C3,** A4, A7, B5, F2	**A5, B4**, B6, A4, A7, B5, F2, G2	**E4, E5, F3**, A4, A7, B5, F2, G2	**E4, H3**, A4, A7, B5, F2, G2	**A5, B4, E5**, A5, A7, B5, B6			
**Myoglobin**	A7, A9, C2, E4	E4	A7, A9, E4	A7, A9, C2	A7, A9, C2	A7, A9, C2, E4	**E7**, A7, A9, E4	**H3, H5**, A7, A9, C2, E4	**F2**, A7, A9, C2, E4	**D9, D11**, A7, A9, E4, C2	**H3, H5, D9, E7**, A7, A9, C2, E4			
**α-lactalbumin**	D4, D3, F5, F7, C10	**B7**, D4, D3, F7,C10	**B9**, D4, D3, F5, F7, C10	D4, D3, F5, F7, C10	D4, D3, F5, F7	D4, D3, F5, F7, C10	**C3**, D4, D3, F5, C10	**H2**, D4, D3, F5, F7, C10	**A3, A5**, D4, D3, F5, C10	**B7, C2, C3**, D4, D3, F5, F7, C10	D4, D3, F5, F7, C10			
**Catalase**	E3, E5, F4, F5, H2	**B5, B7**, E3, E5, H2	E3, E5, F5, H2	E3,E5, F4, F5, H2	E3, E5, F4, F5, H2	E3, E5, F4, F5, H2	**B7, B11**, E3, E5, F4, F5	**B7**, E3, E5, F4, F5	**D5, D7, G7**, E3, F4, F5, H2	**B9**, E3, E5, F4, F5, H2	**B7, D5, D7, B9, B11**, **H5,** E5, H2, E3			
**Xylanase**	A7, A9, G2, H5, H9	**H3**, A7, A9, G2, H5	A7, A9, H5, H9	A7, A9, G2, H5, H9	**C11**, A9, G2, H5, H9	A7, A9, G2, H5, H9	**F4, F5, H7**, A7, A9, G2, H9	A7, A9, G2, H5, H9	**E4, H3, H4**, A7, A9, H5, H9	**D4, D9**, A7, A9, G2, H5	**C11, E4, H3, H4, F4, F5**, A7, A9, G2, H5			
**Thaumatin**	D10, F9, F11, H2	**D5, D7**, F9, F11	F9, F11, H2	D10, F9, F11, H2	**D5**, D10, F9, F11, H2	D10, F9, F11, H2	**D2, E2**, D10, F9, F11, H2	**A2 , A5, D7**, D10, F9, F11	**F3, F5**, D10, F9, F11, H2	**D7, G2**, D10, F9, F11	**A2, A5, D7, G2, E2**, F9, F11, H2			
